# Extra- and Intracranial Cerebral Vasculitis in Giant Cell Arteritis: An Observational Study

**DOI:** 10.1097/MD.0000000000000265

**Published:** 2014-12-02

**Authors:** Delphine Larivière, Karim Sacre, Isabelle Klein, Fabien Hyafil, Laurence Choudat, Marie-Paule Chauveheid, Thomas Papo

**Affiliations:** From the Université Paris Diderot, PRES Sorbonne Paris Cité, Paris, France; Assistance Publique Hôpitaux de Paris, Hôpital Bichat; Département de Médecine Interne, Paris, France (DL, KS, MPC, TP); Département Hospitalo-Universitaire (DHU) Fire, Paris, France (KS, TP); INSERM U1146, Paris, France (KS, TP); Université Paris Diderot, PRES Sorbonne Paris Cité, Paris, France; Assistance Publique Hôpitaux de Paris, Hôpital Bichat; Département de Radiologie, Paris, France (IK); Université Paris Diderot, PRES Sorbonne Paris Cité, Paris, France; Assistance Publique Hôpitaux de Paris, Hôpital Bichat; Département de Médecine Nucléaire, Paris, France (FH); and Université Paris Diderot, PRES Sorbonne Paris Cité, Paris, France; Assistance Publique Hôpitaux de Paris, Hôpital Bichat; Département de Pathologie, Paris, France (LC).

## Abstract

Recognizing giant cell arteritis (GCA) in patients with stroke may be challenging. We aimed to highlight the clinical spectrum and long-term follow-up of GCA-specific cerebrovascular accidents.

Medical charts of all patients followed in a French Department of Internal Medicine for GCA between January 2008 and January 2014 were retrospectively reviewed. Patients with cerebrovascular accidents at GCA diagnosis were included. Diagnosis of GCA was based on American College of Rheumatology criteria. Transient ischemic attacks and stroke resulting from an atherosclerotic or cardioembolic mechanism were excluded. Clinical features, GCA-diagnosis workup, brain imaging, cerebrospinal fluid (CSF) study, treatment, and follow-up data were analyzed.

From January 2008 to January 2014, 97 patients have been followed for GCA. Among them, 8 biopsy-proven GCA patients (mean age 70 ± 7.8 years, M/F sex ratio 3/1) had stroke at GCA diagnosis. Six patients reported headache and visual impairment. Brain MR angiography showed involvement of vertebral and/or basilar arteries in all cases with multiple or unique ischemic lesions in the infratentorial region of the brain in all but one case. Intracranial cerebral arteries involvement was observed in 4 cases including 2 cases with cerebral angiitis. Long lasting lesions on diffusion-weight brain MRI sequences were observed in 1 case. All patients received steroids for a mean of 28.1 ± 12.8 months. Side effects associated with long-term steroid therapy occurred in 6 patients. Relapses occurred in 4 patients and required immunosuppressive drugs in 3 cases. After a mean follow-up duration of 36.4 ± 16.4 months, all but 1 patient achieved complete remission without major sequelae.

The conjunction of headache with vertebral and basilar arteries involvement in elderly is highly suggestive of stroke associated with GCA. Intracranial cerebral arteries involvement with cerebral angiitis associated with long lasting brain lesions on diffusion-weight brain MRI sequences may occur in GCA. Both frequent relapses and steroid-induced side effects argue for the use of immunosuppressive agents combined with steroids as first-line therapy.

## INTRODUCTION

Giant cell arteritis (GCA) is the most frequent vasculitis in patients aged over 50 years old in Europe and North America.^1^

Stroke may occur in patients with GCA. In most cases, stroke is related to vasculitis of extracranial cerebral arteries causing vertebral or internal carotid arteries stenosis.

However, cerebrovascular accidents are rare in the setting of GCA and specific involvement of cranial arteries may be difficult to distinguish from non-specific atherosclerotic lesions in the elderly. The first study addressing that topic reported stroke in 12 (7.2%) out of 166 patients with biopsy-proven GCA. However, the chronology was consistent with a specific association between stroke and GCA in only 4 cases (2.4%).^2^ Further studies reported rates of GCA-associated stroke ranging from 1.5% to 7%^3–7^ with cerebrovascular accidents occurring in 8 (2.8%) of the 287 patients with biopsy-proven GCA in the largest population based-study published so far.^8^

The aim of our study was to highlight the clinical spectrum and long-term follow-up of GCA-specific stroke. Specifically, we analyzed, the cerebral arteries specific involvement—both extra- and intracranial—and the pattern of associated ischemic brain lesions in 8 biopsy-proven GCA patients with stroke at GCA diagnosis.

## METHODS

### Patients

All adult patients followed in a French Department of Internal Medicine (Bichat University Hospital, Paris, France), who had a GCA between January 2008 and January 2014, were retrospectively screened. Patients with stroke at GCA diagnosis were studied. Only patients with GCA defined according to American College of Rheumatology criteria were included.^9^ Strokes were classified according to their clinical features and confirmed by cranial computer tomography (CT) scan and magnetic resonance imaging (MRI). Patients were excluded in case of transient ischemic attacks (symptoms self-limited within less than 24 hours and without residual neurological damage, neither lesion on cerebral imaging). Atherosclerosis or embolic stroke was discarded in all cases.

### Data Collection

We used a standardized data collection form for all cases, including demographic data (sex and age), date of GCA diagnosis (i.e. date of temporal artery biopsy [TAB]), traditional cardiovascular risk factors (tobacco use, high blood pressure, diabetes, and hypercholesterolemia), clinical (fever, headache, jaw claudication, scalp hyperesthesia, cough, polymyalgia, visual loss, abnormal temporal artery on physical examination, and neurologic examination), biological (c-reactive protein [CRP] blood level and/or erythrocyte sedimentation rate), imaging (cranial MRI, vascular ultrasound study, MR angiography, and/or CT angiography, *fludeoxyglucose* [FDG] F 18 positron emission tomography [PET]) data, TAB results, treatment, and outcome.

### Ethics Statement

Our study is a human non-interventional study where (1) subjects were not assigned to treatment, (2) subjects were assigned to a diagnosis strategy within current practice, (3) epidemiological methods were used to analyze the data, and (4) information used in the study were collected for clinical care. According to the Public Health French Law (art L 1121-1-1, art L 1121-1-2), approval from Institutional Review Board and written consent are not required for human non-interventional studies. For ethical consideration, patients were however informed that data that was collected in medical records might be used for research study in accordance to privacy rule. The study protocol conforms to the ethical guidelines of the 1975 Declaration of Helsinki.

## RESULTS

From January 2008 to January 2014, 97 consecutive patients have been followed for GCA. Among them, 12 patients with a stroke at GCA diagnosis were identified. One case was excluded because atrial fibrillation was obviously the cause for ischemic brain lesions. Three cases were excluded because GCA diagnosis did not satisfied ACR criteria when retrospectively reviewed. Eight biopsy-proven GCA patients (mean age 70 ± 7.8 years old; M/F sex ratio 3/1) were studied. The mean time elapsing between stroke diagnosis (assessed by cranial MRI) and GCA diagnosis (confirmed by TAB results) was of 7.7 + 7.0 (range 2–20) days. Detailed clinical characteristics are given in Table 1.

**TABLE 1:**
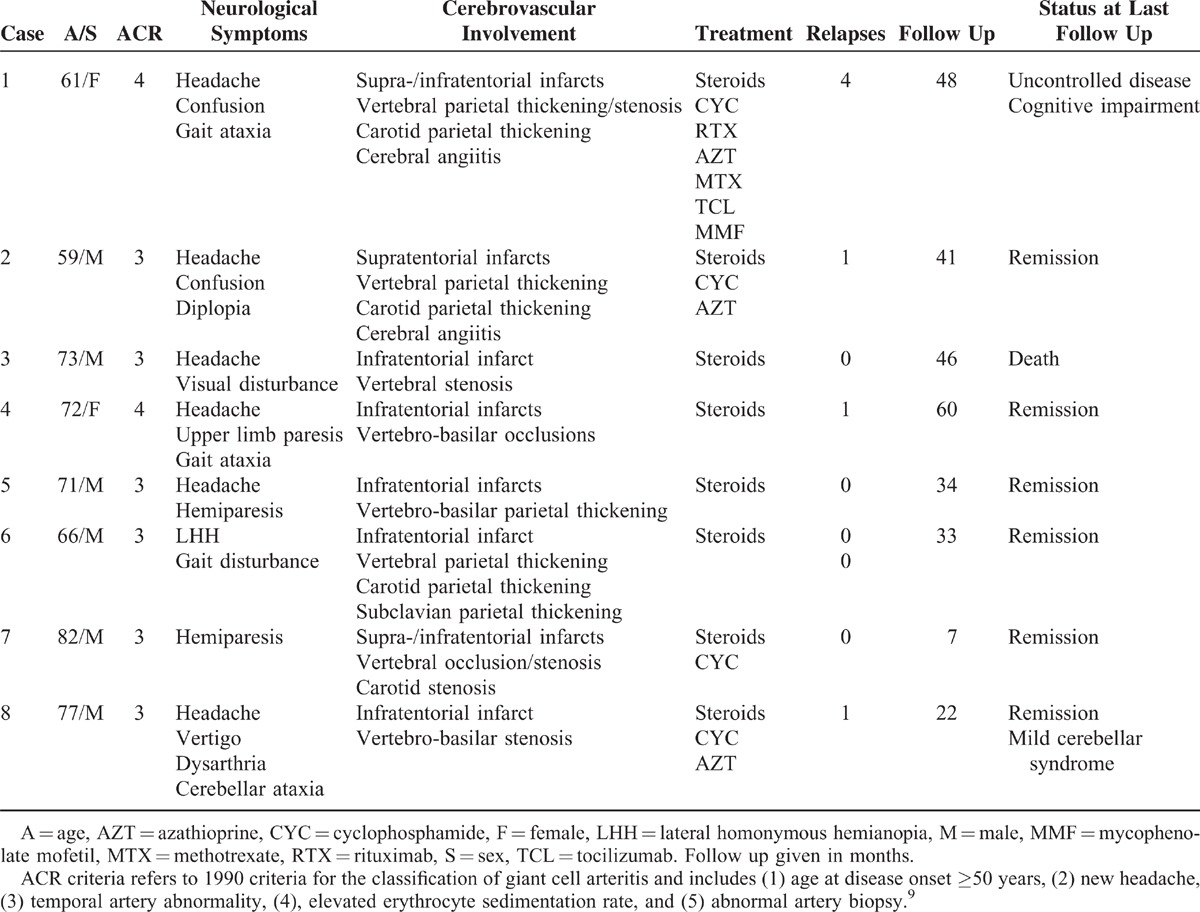
Patients’ Characteristics

### Case 1

**FIGURE 1:**
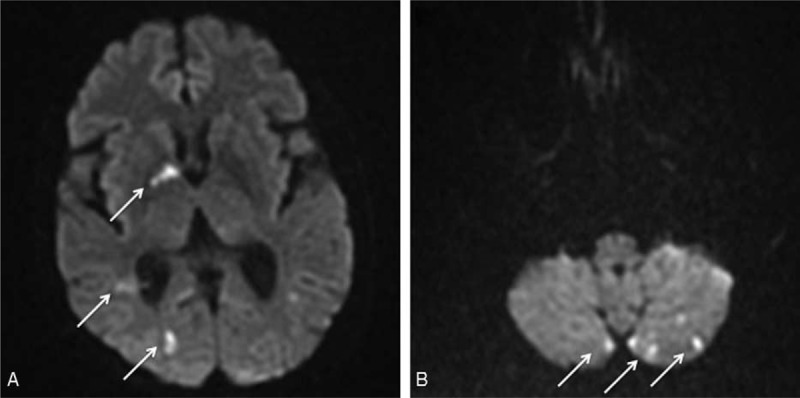
Diffusion-weighted images of cranial MRI. Acute hemispheric (A) and cerebellar (B) ischemic lesions.

**FIGURE 2:**
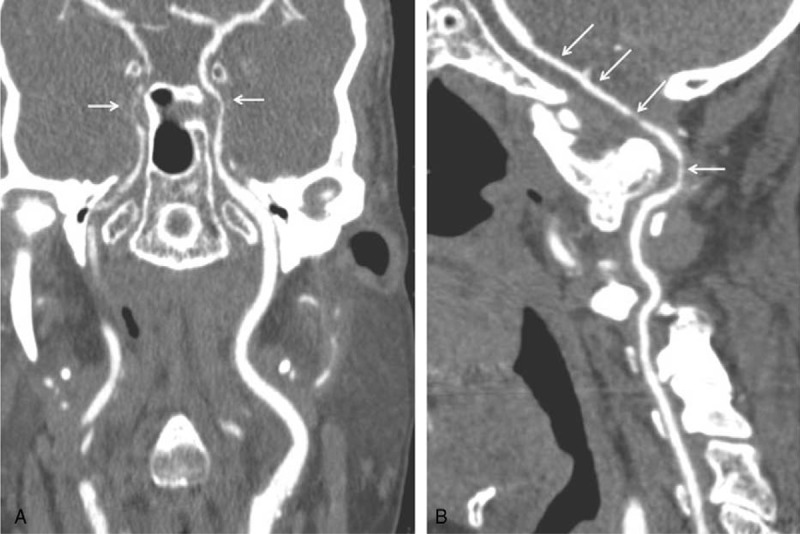
Cranial CT angiography in multiplanar reconstruction. Long, regular, circumferential stenosis of the cavernous portion of both carotid arteries (A). Regular narrowing of the V3 and V4 segments of the vertebral arteries (B).

A 61-year-old woman was admitted in October 2009 for confusion, headache, jaw claudication, and blurred vision. She had a past history of hypercholesterolemia, smoking, and sleep apnoea syndrome. Clopidogrel was taken for an asymptomatic right internal carotid stenosis. Physical examination revealed symptoms suggestive of frontal lobe syndrome and gait ataxia. Right temporal artery was tender. CRP blood level was 42 mg/L. Analysis of cerebrospinal fluid (CSF) showed a raised protein level (0.72 g/L) with no cells. Ophthalmologic examination displayed left optic neuritis. Cranial MRI showed multiple recent hemispheric and cerebellar small infarcts (Figure 1). The MR and CT angiography (Figure 2) revealed circumferential parietal thickening of the carotid siphons along with bilateral and multiple stenosis of the intra- and extracranial portions of the vertebral arteries. Positron emission tomography showed high FDG uptake in the terminal portion of the vertebral arteries, carotid siphons, and femoral arteries. The diagnosis of GCA was confirmed by a TAB showing inflammatory cellular infiltrate with giant cells in the media and interruption of elastic lamina. Prednisone (1 mg/kg/day) and aspirin were started and clinical status improved.

In August 2010, right facial paralysis, confusion, and marked ataxia occurred while the patient was still receiving 17.5 mg/day of prednisone. CRP level was 10 mg/L. Cranial MRI showed multiple new ischemic lesions. Cardiac ultrasound study and 24 h ECG monitoring were normal. Vasculitis relapse was considered. High-dose methylprednisolone pulses and intravenous cyclophosphamide were started.

In December 2010, headache, dysarthria, ataxia, amnesia, and frontal lobe syndrome recurred. C-reactive protein blood level was 28 mg/L. Cranial MRI showed new bilateral small ischemic lesions and obvious worsening of the right vertebral artery stenosis. Three intravenous pulses of methylprednisolone and rituximab (1 g Day 1 and Day 15) were administered.

In February 2011, a third relapse—with cognitive impairment, raised CRP blood level at 53 mg/L, and new ischemic lesions on cranial MRI—occurred under 30 mg/day of prednisone. Azathioprine was started.

In August 2012, a sudden right hemiparesis was caused by a new left frontal ischemic lesion seen on brain MRI. CRP level was 250 mg/L. Tocilizumab was started but stopped after 4 perfusions because of a neutropenia. Eventually, mycophenolate mofetil was initiated. Four years after diagnosis the patient was still receiving 15 mg/day of prednisone and suffers severe cognitive impairment.

### Case 2

A 59-year-old man was hospitalized in January 2011 for a 1-month-history of weight loss, confusion and fever. The patient suffered intense headache, jaw claudication, scalp dysesthesia, cough, intermittent diplopia, and amaurosis. He was a heavy smoker and had a history of hypertension and hyperlipidemia. Physical examination was otherwise normal. CRP blood level was 56 mg/L. CSF showed lymphocytic meningitis (18 cells/mm^3^ with 68% of lymphocytes, protein level 0.43 g/L). Brain MRI showed 4 small acute ischemic lesions, in the caudate nucleus, the right internal capsule, and the lenticular nucleus. Magnetic resonance angiography disclosed circumferential and enhanced parietal thickening of extra- and intracranial carotid and vertebral arteries. Giant-cell arteritis was suspected. Pulses of methylprednisolone and aspirin were administrated leading to dramatic clinical improvement. TAB showed giant cells concentrated near the internal elastic lamina and mononuclear cells infiltrate in the media and adventitia.

Six months later, he was hospitalized for headache, left hemidysesthesia, and horizontal diplopia while still receiving 15 mg/day of prednisone. CRP blood level was 6 mg/L. Cranial MRI showed a new left frontal ischemic lesion without any further arterial stenosis on MR angiography. Cardiac ultrasound and 24 h ECG monitoring were normal. Relapse was considered and treated with intravenous methylprednisolone pulses and cyclophosphamide. After 6 months, cyclophosphamide was switched for azathioprine. Azathioprine and prednisone were stopped in January and July 2014, respectively.

### Case 3

A 73-year-od man was admitted in December 2008 for transient blurred vision. Past medical history included lower limbs arteritis, coronary bypass, mesenteric artery bypass, and asymptomatic left carotid artery stenosis. He was treated for hypertension and hyperlipidemia and received a daily dose of clopidogrel. Cranial MRI revealed a right cerebellar peduncle stroke with stenosis of the ostium and V4-segment of the left vertebral artery. The ophthalmologic exam was normal. CRP blood level was 14 mg/L. Aspirin was started and a surgical vertebral artery-common carotid artery transposition was carried out.

Five months later, the patient displayed headache and swelling of the temporal arteries. On examination, both temporal arteries were tender without right temporal pulse. Cranial MR angiography revealed new multiple stenosis of the V2-segment of the left vertebral artery. TAB confirmed GCA diagnosis by showing numerous aggregates of giant cells in the media related to point of interruption of elastic lamina.

Oral prednisone treatment (0.7 mg/kg/day) was started. Complete clinical remission occurred and prednisone was stopped in March 2012 without relapse during follow-up. The patients died from pancreas adenocarcinoma in October 2012.

### Case 4

A 72-year-old woman was hospitalized in June 2009 for left upper limb paresis and gait ataxia. She had a medical history of hypertension. She complained of weight loss, headache, jaw claudication, scalp hyperesthesia, and left amaurosis. Right temporal artery pulse was decreased. CRP blood level was 185 mg/L. Cranial MRI revealed bilateral cerebellar infarcts. Doppler ultrasound showed occlusions of V4-segments of both vertebral arteries and basilar artery. Fundoscopy was normal. CSF analysis showed lymphocytic meningitis (21 elements with 86% of lymphocytes, protein level 0.87 g/L). TAB showed a thickened intimal fibrosis and mononuclear cells infiltrate in the media and adventitia. Giant cells were seen in the media.

Methylprednisone pulses and aspirin were started. Three months later, occlusions of both vertebral arteries were still present on MR angiography despite complete clinical remission. Prednisone was eventually stopped in August 2011.

In July 2013, GCA relapsed with polymyalgia and weight loss. CRP blood level was 123 mg/L. At this time, PET showed mild FDG uptake involving thoracic and abdominal aorta, supra-aortic arteries, shoulders, and hips. Prednisone treatment was resumed at 40 mg per day. Clinical situation promptly improved and prednisone dose was slowly tapered.

### Case 5

**FIGURE 3:**
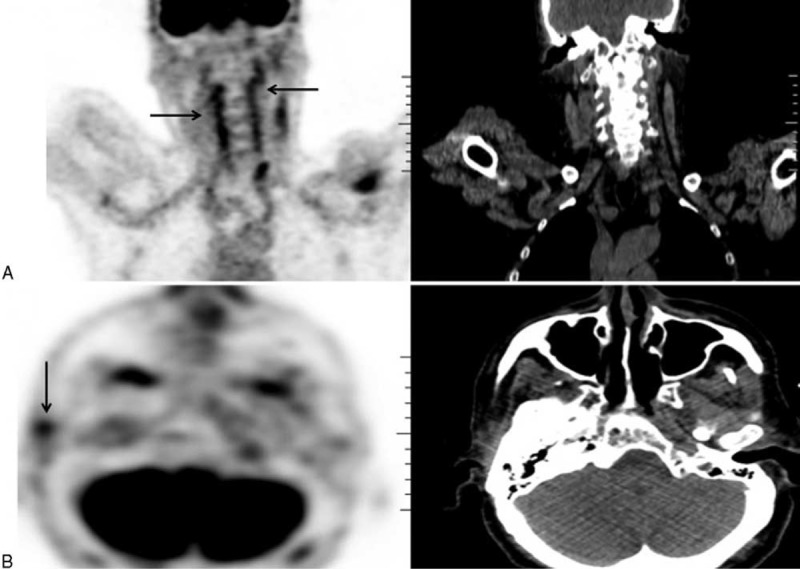
Coronal PET with CT scan. Intense 18-FDG uptake of both vertebral arteries (A) and external carotid arteries branches including temporal artery (B).

A 71-year-old man was hospitalized in March 2011 with a 2-month history of cervical pain, fever, weight loss, and cough. He had a medical history of hypertension and prostatectomy for adenocarcinoma in 2009. Three days after admission, a right hemiparesis occurred. CRP level was 113 mg/L. Cranial MRI showed a left paramedian pontine infarction. Five days later, a second brain MRI disclosed a new right cerebellar ischemic lesion. Magnetic resonance angiography revealed a regular and circumferential thickening of basilar and vertebral arteries with gadolinium arterial wall enhancement. Positron emission tomography showed an FDG uptake in both vertebral arteries and external carotid arteries branches (Figure 3). TAB showed a thickened intima with a neutrophils infiltrate in the media and mononuclear cells in adventitia. One giant cell was present near a break in the internal elastic lamina. GCA diagnosis was confirmed and prednisone was started at 1 mg/kg/day.

In May 2011, the patient suffered transient confusion. Brain MRI found 2 new ischemic lesions. CRP blood level was 13 mg/L. Because the patient was considered in clinical remission, close monitoring was undertaken and treatment was not intensified. Prednisone was eventually stopped in June 2013. In January 2014, the patient was in clinical remission without treatment.

### Case 6

**FIGURE 4:**
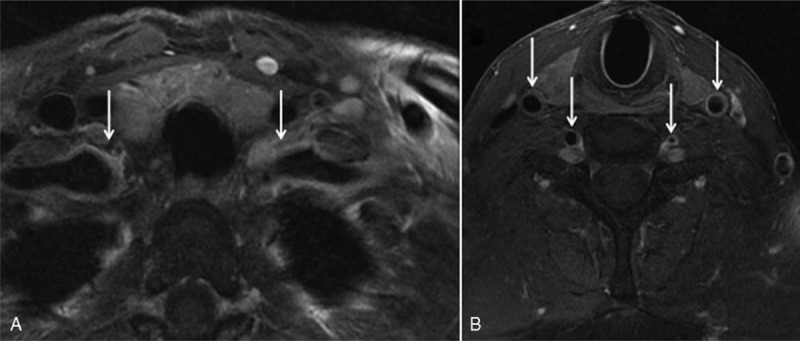
Cranial MR angiography. Enhanced thickening of subclavian (A), vertebral and common carotid (B) arteries.

A 66-year-old man was admitted in October 2011 for gait disturbance and right lateral homonymous hemianopia. He had hypertension and obesity. CRP blood level was 87 mg/L. Cranial MRI disclosed a left paramedian pontine infarct. Positron emission tomography showed a diffuse FDG uptake of subclavian, vertebral, internal carotid arteries, and thoraco-abdominal aorta. Magnetic resonance angiography study showed a circumferential thickening and gadolinium arterial wall enhancement of the subclavian arteries, the vertebral arteries, and the common carotid arteries (Figure 4). TAB specimen microscopic analysis showed a neutrophils and mononuclear cells infiltrate in the media with small breach of the internal elastic lamina.

Prednisone (0.7 mg/kg/day) and clopidogrel were started. Clinical remission was obtained. Steroid treatment was eventually stopped in April 2013.

### Case 7

**FIGURE 5:**
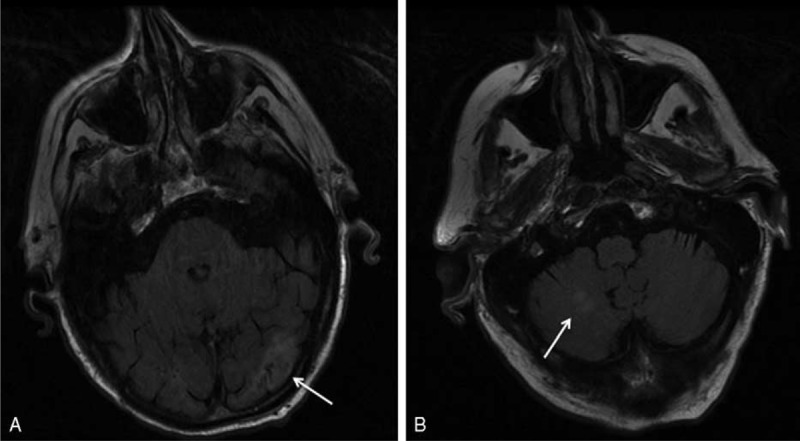
Cranial MRI. Left occipital (A) and right cerebellar (B) ischemic lesions.

In January 2014, an 82-year-old man was referred in our department for sudden blindness and scalp hyperesthesia. He had no cardiovascular risk factors besides a smoking history. Physical examination showed a left hemiparesis. Left temporal pulse was absent. Fundoscopy confirmed a left acute anterior ischemic optic neuropathy. The right optic disc was pale. CRP blood level was 84 mg/L. Cranial MRI and MR angiography showed multiple and bilateral small ischemic lesions, a right frontal and left cerebellar infarction (Figure 5) and occlusion of the right vertebral artery. CT angiography showed multiple stenosis on both vertebral, common carotid, and internal carotid arteries. TAB showed an intimal fibrosis that slightly reduced the lumen of the artery. The media was infiltrated by mononuclear cells, neutrophils, plasmocytes, and giant cells. Internal elastic lamina was ruptured.

High-dose intravenous methylprednisolone pulses followed by oral prednisone, intravenous cyclophosphamide, and aspirin were started. CRP blood level normalized within 8 days and no further neurological event was observed.

### Case 8

A 77-year-old man was admitted in September 2012 for prominent fatigue, headache, scalp dysesthesia, and jaw claudication. He was treated for hypertension. He had recently suffered 2 transient episodes of vertigo and dysarthria. Physical examination revealed a left cerebellar ataxia. CRP blood level was 61 mg/L. MRI showed a left cerebellar ischemic lesion with both vertebral and basilar arteries stenosis. TAB specimen microscopic analysis showed intimal fibrosis with focal lymphocytic aggregates in adventitia. Oral prednisone (0.7 mg/kg/day) and aspirin were started resulting in dramatic clinical and biological improvement.

In January 2013, left hemiplegia and cerebellar syndrome recurred while the patient was under 40 mg per day of prednisone. CRP blood level was 36 mg/l. Brain MRI with MR angiography showed a right laterobulbar and cerebellar ischemia with gadolinium arterial wall enhancement in both vertebral arteries.

High-dose intravenous methylprednisolone and cyclophosphamide pulses were started. After 6 months, cyclophosphamide was switched for azathioprine. Under azathioprine and low dose of prednisone, the patient was in sustained clinical and biological remission. Left cerebellar ataxia was mild and no relapse occurred.

### Biological and Histological Findings

At diagnosis, CRP ranged from 14 to 185 mg/L (mean of 80 ± 52 mg/L). Of note, previous studies suggested that a strong acute-phase response in GCA could be associated with a low risk for ischemic events.^3,4,8^

A lumbar puncture was performed in 5 cases. CSF analysis was abnormal in 4 cases showing a mild hyperproteinorachia (cases 1, 4, and 7), and/or lymphocytic meningitis (cases 2 and 4). Interestingly, CSF abnormalities at diagnosis were found in 3 of the 4 patients who relapsed during follow-up.

Temporal artery biopsy (TAB) showed arteritis in all patients, with giant cells arteritis in 6 cases. Of note, in one patient (case 8), specimen analysis only showed intimal fibrosis and focal lymphocytes aggregates in adventia with no disruption of the internal elastic lamina nor infiltrate cells in the media layer. It has been suggested that histological features such as aggregates of giant cells, presence of plasmocytes, intimal thickening, neovascularization, and arterial occlusion could be associated with ischemic complications associated with GCA.^10,11^

### Imaging Findings

At diagnosis, cranial MRI showed multiple (n = 5) or unique (n = 3) ischemic lesions that involved the infra-tentorial region of the brain in all but one case. During follow-up, long lasting brain high-intensity lesions in diffusion-weighted imaging were observed in one patient (case 1). In this case, cranial MRI performed 105 days after the first relapse disclosed new high-intensity lesions in semioval centers despite no obvious GCA activity. Of note, high-intensity lesions were still present 60 and 120 days later when the patient relapsed for the second and third time, respectively.

Brain MR angiography was abnormal in all cases and showed mural thickening (cases 1, 2, 5, and 6), stenoses (cases 1, 3, and 8), or occlusion (patients 4 and 7) of cranial arteries. Vertebral and/or basilar arteries were involved in all cases. Intracranial cerebral arteries were involved in 4 patients (cases 1, 2, 4, and 8) included 2 cases (cases 1 and 2) of clear-cut cerebral angiitis. Mural thickening was enhanced after injection of gadolinium in all but 1 case (cases 2, 5, and 6). Conventional cerebral angiography was performed in 2 patients at diagnosis (cases 3 and 6) but had no additive value.

Brain MR angiography was performed during follow up in 3 patients (cases 3, 4, and 8). Arterial lesions observed at diagnosis did not improve overtime whereas clinical outcome was good in all cases. In one patient (case 3), a bulge appeared alongside with the left vertebral stenosis on MR angiography performed 27 months after GCA diagnosis.

PET was performed in 6 patients. When performed at CGA diagnosis and before steroids treatment, increased vascular FDG uptake was observed in all cases (cases 1, 5, and 6). FDG uptake involved terminal portion of the vertebral (n = 3), internal carotid (n = 2), external carotid (n = 1), subclavian (n = 1), femoral (n = 1) arteries, and aorta (n = 1). Standardized uptake value ranged from 3 to 7.2.

PET was negative in all steroid-treated patients at initial GCA presentation (cases 2 and 7) or relapse (cases 1, 2, and 8).

### Treatment

All patients were treated with oral prednisone initiated at a daily dose of 0.7 to 1 mg/kg. Intravenous methylprednisolone pulses were also administered at GCA diagnosis (n = 3) or relapse (n = 2). The mean duration of steroid therapy was 28.1 ± 12.8 months. Side effects associated with steroid therapy—including glaucoma (n = 1), osteonecrosis (n = 2), osteoporotic fracture (n = 1), diabetes (n = 2), myopathy (n = 2), and psychiatric disorders (n = 2)—occurred in 6 patients during follow-up. Immunosuppressant drugs were used in 4 patients (cases 1, 2, 7, and 8) who all received intravenous cyclophosphamide monthly for 6 months as induction therapy, followed by remission maintenance treatment (Table 1). In 3 cases (cases 1, 2, and 8), immunosuppressant drugs were used because of a refractory disease. Antiplatelet therapy was added to steroids in all but one case.

### Outcome

Mean follow-up was 36.4 ± 16.4 months. Cerebral disease recurred in 4 patients (cases 1, 2, 4, and 8) during follow up. In 3 cases, (cases 1, 2, and 8) relapse occurred between 4 and 9 months after GCA diagnosis while patients were still receiving high dose of prednisone. Of note, CRP blood level could be normal at relapse (cases 1 and 2). All but 1 patient eventually achieved complete remission without major sequelae. One patient (case 1) had refractory vasculitis and developed severe vascular dementia. One patient (case 3) died from pancreas cancer 4 years after GCA diagnosis.

## DISCUSSION

We report on extra- and intracranial cerebral vasculitis in GCA.

In our series, traditional cardiovascular risk factors were found in all patients including high blood pressure (n = 7), smoking history (n = 5), hypercholesterolemia (n = 3), and diabetes (n = 1). In addition, 2 patients were receiving platelet antiaggregant therapy for atherosclerosis disease. Previous reports suggested that traditional cardiovascular risk factors such as tobacco use, hypertension, and past history of ischemic heart disease were associated with the occurrence of stroke in patients with GCA.^4,8,12^ Antiaggregant therapy has been advocated to decrease the risk for severe ischemic complications in GCA.^13,14^ Cardiovascular risk factors are frequent in the elderly and, although known to confer a risk for atherosclerosis disease, no clear evidence exists to connect traditional risk factors with GCA-specific stroke. In our patients, the fact that stroke occurred shortly after GCA symptoms onset, clearly argues for a link between vasculitis and ischemic brain lesions. Moreover, the pattern of vascular lesions—mostly vertebral and basilar arteries involvement—observed in our series was clearly distinct from usual atherosclerosis lesions. Of note, we excluded one case of embolic stroke caused by atrial fibrillation in a patient with biopsy-proven GCA.

The male predominance observed in our series of patients with GCA-specific stroke contrasts with the usual female predominance in GCA and has already been reported.^7,8^

The most frequent symptoms associated with GCA were headache (n = 6) and visual impairment (n = 6). Headache, while very common in GCA, may be a powerful clue in patients with stroke. Indeed, in a study comparing bilateral vertebral arteries occlusion associated with GCA or atherosclerotic disease, GCA patients displayed new headache in all cases contrasting with only 22% of headaches reported in cases associated with atherosclerotic disease.^15^ Concomitant visual loss caused by anterior ischemic optic neuropathy also argues for GCA-specific stroke.^8^

Bilateral distal involvement of the vertebral and basilar arteries was observed in all patients in our series, contrasting with atherosclerosis which involves more frequently carotid branches arteries.^16^ Such an intriguing feature has extensively been reported by others.^6,8,17^

Cerebral angiitis stands as an exceedingly rare, almost controversial complication of GCA. The decreased amount of elastin present in intradural vessels—as macrophages appear to target elastin—was postulated to explain the relative sparing of the more distal vessels in GCA. In previous reported series, including series with post-mortem examinations,^17–19^ only 2 cases of GCA with cerebral angiitis have been reported.^20^ Interestingly, 2 of 8 patients (cases 1 and 2) showed cerebral angiitis in our series. Although neither brain biopsies nor conventional cerebral angiography was performed, the diagnosis of cerebral angiitis was based in both cases on neurologic manifestations including step-wise cognitive decline, CSF features, and imaging findings consistent with medium and small-sized intracranial vessels involvement. Interestingly, in both cases, cerebral angiitis relapsed without obvious progression of extracranial lesions.

Although regression of arterial stenosis upon prednisone has been reported,^21–23^ such was never the case in our series with a 36.4 ± 16.4 months follow-up, even when clinical outcome was good. In one case, long-term MRI follow-up has suggested that vertebral artery involvement may eventually cause aneurysm, as described for aorta in GCA. On brain MRI follow-up, high-intensity lesions in diffusion-weighted imaging could last more than 6 months, which strongly contrasts with the progressive fading of ischemic lesions usually observed over weeks in atherosclerosis stroke. Since cerebral angiitis was highly refractory in that case, long-lasting high-intensity lesions in diffusion-weighted imaging may have a bad prognostic significance in GCA-specific stroke.

All patients received steroid therapy complicated with frequent side-effects. Four patients (50%) experienced a relapse. In 3 cases, relapse occurred shortly after diagnosis in patients receiving more than 15 mg/day of prednisone. Such an outcome contrasts with previous studies in general biopsy-proven GCA population where relapse usually occurs in patients receiving less than 10 mg/day of prednisone.^24^ Because of steroids side-effects and frequent relapses, immunosuppressive treatment may be considered early in GCA-related stroke. Antiplatelet therapy, although controversial, may help to prevent severe ischemic complications associated with GCA.^13,14^ The potential role, if any, for endovascular treatment remains to be defined.

Stroke is reported to be a cause of death in GCA patients.^17,20,25–29^ In our series, no patient died from GCA-specific stroke. A good clinical outcome was observed in most patients, maybe because of early steroid therapy. Of note, the mortality rate in GCA patients from population-based cohort studies appears to equal that of general population.^29,30^

Our study has limitations since it is a monocentric retrospective analysis of a small number of patients. Moreover, because the GCA population was not analyzed as a whole, our study gives no information regarding the risk factors for stroke in GCA patients.

In conclusion, we confirm that the conjunction of headache with stroke caused by bilateral vertebral and basilar arteries involvement in the elderly is highly suggestive of GCA. Cerebral angiitis associated with long lasting high-intensity brain lesions in diffusion-weighted imaging on MRI may also occur in GCA. Both relapses and steroid-induced side effects support the need for immunosuppressive drugs as first-line therapy in GCA-specific stroke.
